# Clinical Cases in the Mercy Hospital, Involving Obscure Nervous and Cerebral Symptoms

**Published:** 1874-12-15

**Authors:** N. S. Davis


### MEDICAL EXAMINER.

A

Semi-Monthly Journal of Medical Sciences.

EDITED BY N. S. DAVIS, M.D., AND F. H. DAVIS, M.D.

No. XXIV. CHICAGO, DEC. 15, 1874. VOL. XV.

### Original Communications.

### CLINICAL CASES IN THE MERCY HOSPITAL, INVOLVING OBSCURE NERVOUS AND CEREBRAL SYMPTOMS, AND CONSTITUTING THE SUBJECT OF A CLINIC BY PROF. N. S. DAVIS.

CASE I.—R. M——, a native of Ireland, aged forty-seven years; married; medium size, well-proportioned, and accustomed to work in the stone-quarries for the last fifteen years; was admitted into the medical wards of the hospital yesterday.

*History.*—He states that while at his accustomed work in the stone-quarry, during a hot day in summer, *fourteen* years since, he had a partial *sunstroke*, sufficient to nearly interrupt consciousness for a few hours. He recovered sufficiently to resume his work in a few days, but there has remained a numbness and a sense of tightness in the scalp over the top of the head, ever since. He describes it as a feeling that prompts him to frequently rub the scalp and pull the hair, and inspection shows that along the sagital suture, from the anterior to the posterior fontanelle, the hair looks worn off and the skin thickened by long-continued rubbing. With the exception of a few attacks of ague his general health continued sufficiently good to enable him to follow his laborious occupation with little or no interruption until within the last year.

About one year since he began to feel a sense of heat and burning in the scalp over the top of his head, with occasional sudden shocks, like slight electric currents, through the face and head. During the last three months this burning in his head has become constant, and his appetite and strength have steadily declined.

*Present Condition.*—Expression of countenance anxious and care-worn; lips dry; pupils small; skin generally rather dry and harsh; tongue thin, a little red at the tip and edges, with a thick whitish coat over the upper surface; pulse 90 per minute and soft; body temperature 100° F.; urine scanty and rather red; bowels inactive; some thirst but no appetite; respiratory movements variable but not hurried; no cardiac or respiratory sounds of an unnatural character; and walks like one afraid of falling. He now complains constantly of a terrible burning pain in the top of his head; burning sensations in the front part of his legs, below the knees, especially at night; some muscular twitchings; inability to sleep; and once or twice each day he gets a violent shock which he says "knocks him down entirely." He describes this shock as a *rush* from his chest upward through his neck and face to the top of his head, accompanied by a partial suspension of consciousness, compelling him to lie down immediately or fall. He claims not to lose his consciousness entirely, nor to have any well-marked spasmodic action of the voluntary muscles. The severe symptoms of shock last but a few moments, but he remains weak, timid, and complaining severely of the pain in his head three or four hours. I cannot learn that these paroxysms are preceded or accompanied by any indications of chilliness, or that they come at any regular hours, although he says they are most frequent in the mornings and evenings.

*Pathology.*—What are the pathological conditions involved in this case? That the assimilative, excretory, and nervous functions are in a morbid or generally disturbed condition, is very evident; yet neither the state of the circulation nor the temperature of the patient indicate any form of idiopathic fever. It is probable that the loss of appetite, the coated tongue, the general tardiness of secretion, have resulted from the long-continued and increasing disturbance of the nervous functions, and the consequent mental anxiety and want of sleep. But what is the nature of the nervous derangement from which he suffers? Do those derangements depend upon disease within the cranium; and if so, what is the nature of such disease? The numbness, or anæsthetic condition of the top of his head, continuing since the partial sunstroke fourteen years since; the very gradual manner in which all his present symptoms have been developing during the past year, as well as the character of the present symptoms, incline me to think there is some important change in the condition of some portion of the cerebral mass. It is probable that certain parts of the brain have been morbidly excitable since the first severe effects of the high temperature fourteen years since; and that such morbid excitability has been gradually modifying the nutrition, as well as steadiness of functional action, until during the last few months it has developed almost constant morbid sensations in his head, with paroxysms steadily increasing in frequency and approximating more nearly to the character of epilepsy.

*Prognosis.*—If we have rightly apprehended the nature of his case the prospect of his recovery is not good.

It is probable that the long-continued morbid sensitiveness has led to more or less molecular or structural change in the superior part of the hemispheres of the cerebrum. And yet, it may be that the more recently established burning sensations in the top of the head, and in the legs, with the muscular twitchings and more violent shocks, as well as the marked general derangement of the secretions, are owing to the supervention of a low grade of inflammatory action in the brain, which might be overcome by judicious and persistent treatment. One of the most discouraging symptoms is the timid, almost childish condition of the patient's mind. It not only adds to the probability of structural lesion, but it constitutes a great obstacle in the way of securing regularity and persistence in carrying out either hygienic or medical treatment.

*Treatment.*—To remove, as far as possible, everything that might act unfavorably on the nervous centres, the patient was directed to have a diet consisting of milk, farinaceous articles, and fruit: carefully avoiding all meat and drinks of an exhilarating character, such as tea, coffee, and all fermented and distilled drinks containing any proportion of alcohol. To correct the secretions, allay nervous excitability, and check the tendency to epileptiform shocks or paroxysms, the following prescription was ordered for the patient:

| | | |
|---|---|---|
| ℞ | Bromide Sodium, | ʒ vi. |
| | Iodide " | ʒ iji. |
| | Bi-Chloride Hydrarg., | gr. i. |
| | Fl. Ext. Con., | ℥ ss. |
| | Tinct. Digitalis, | ℥ i. |
| | Syrup. Prunus. Virg., | ℥ iiiss. |

Mix. Give 1 teaspoonful before each meal, and at bedtime, in a little water.

In further illustration of the effects of high temperature, or partial sunstroke, in producing protracted functional disturbance, the lecturer related the following case:

CASE II.—A laborer, aged twenty-two years, native of Ireland. While engaged in out-door work, in July, 1870, was exposed to the direct rays of the sun on an unusually hot day. The heat produced sufficient effect to cause sudden prostration, with partial loss of consciousness, from which he said he soon recovered so far as to be able to get up and go about the house. But he remained weak; easily excited, timid in feeling, and wholly incapable of exposing himself to the direct rays of the sun without immediately inducing such a feeling of tremulousness or agitation and giddiness, as to impress him with the idea that he would fall unconscious. Nearly two years had elapsed after the attack when the lecturer was first called to the patient. He was sitting in his room with a soft wool hat drawn over his head; his face pale; expression sad and anxious; pulse soft, quick, and 90 per minute; respiration quiet and slow when undisturbed, but short and hurried when making any exertion; temperature natural; urine free; tongue clear; bowels slightly costive and appetite variable, with some impairment of digestion. He complained of muscular weakness and great weariness from slight exertion, but was free from pain and comfortable while quiet and in the shade. If the rays of the sun were allowed to strike him, however, he immediately became excited, complained of very strange and distressing feelings in his head, looked pale, breathed as if tired, and insisted that he would fall over or lose consciousness. And no persuasion or assurances could induce him to venture out uncovered while the sun was shining. His sleep at night was unrefreshing and often disturbed by dreams. He had done no work since his first attack, and had suffered but little loss of flesh.

This case presented no numbness, no nervous or muscular twitchings, and except the pale, anxious and timid expression of countenance, looked as though he might step out and go to his work.

He had consulted several physicians, and claimed to have taken a variety of tonics and stimulants, with little or no benefit. Acting upon the idea that high temperature increases the susceptibility or inherent excitability, and diminishes the affinity of the organic atoms or cells for each other, and regarding the present condition of the patient as a continuance of this disturbed condition of the elementary properties of the cerebral mass, a plan of treatment was adopted, having special reference to the allaying of such morbid susceptibility and the restoration of a healthier degree of tonicity. He was put on a plain nutritious diet; tea, coffee, tobacco, and all alcoholic exhilarants were prohibited, and the following medicines directed to be taken steadily for three or four weeks:

| | | |
|---|---|---|
| ℞ | Tinct. Digitalis, | ℥ j. |
| | Fl. Ext. Scutellaria, | ℥ ii. |
| | " Conium, | ℥ ss. |
| | Syrup Prunus. Virg. | ℥ ss. |
| | Bromide Potassium, | ʒ iv. |

Mix. Take 1 teaspoonful before each meal, and at bedtime. Also

| | | |
|---|---|---|
| ℞ | Oxide of Zinc, | ʒ jss. |
| | Ergotin, | 30 grs. |
| | Ext. Cannabis Ind. | 15 grs. |

Mix. Divide into 40 pills. Take one pill half an hour after breakfast and dinner each day.

Wishing to gain also the invigorating effects of mental courage, hope, and cheerfulness, he received the most positive assurances that he would get well, and that he must not fail to go out-door some every day.

This last part of the programme was by far the most difficult of execution; and it was not until three or four weeks had elapsed under reiterated assurances that he gained sufficient courage and steadiness to venture out with any degree of freedom. He finally recovered, however, and when last heard from had resumed his ordinary work. Two or three other cases of less severity, arising from the same cause, were alluded to as having come under the lecturer's observation during the past year. They were treated on the same principles with satisfactory results, special attention having been given in each case to mental courage and confidence.

---

ACCIDENTAL NEPHROTOMY.—The *Wiener Med. Woch.* states that a man of twenty-five, having been stabbed in the left renal region, a fleshy tumor was extruded through the wound by the act of coughing. This was found to be the kidney, which was eventually removed after a double ligature had been applied to the pedicle. The man did well.—*London Lancet.*

---

"THE PARIS MEDICAL RECORD" is the title of a new venture in medical journalism. The editor states his conviction that, "notwithstanding the great number of medical periodicals in existence, the thirst for knowledge is so intense that the supply is still far short of the demand." Hence this very promising bi-monthly review of the progress of medicine. The journal is published in Paris in the English language, and the initial number is made up mainly of translations of lectures and other contributions to medical science by Paris professors.

